# From lab to field: Innovative RPA‒CRISPR/Cas12a platform for early short-beak and dwarfism syndrome virus nucleic acids detection

**DOI:** 10.1016/j.psj.2025.105191

**Published:** 2025-04-19

**Authors:** Xiuqin Chen, Shizhong Zhang, Su Lin, Meiqing Huang, Shilong Chen, Shao Wang

**Affiliations:** a Institute of Animal Husbandry and Veterinary Medicine, Fujian Academy of Agricultural Science, Fuzhou, Fujian 350013, PR China; b Fujian Animal Diseases Control Technology Development Center, Fuzhou, Fujian 350013, PR China

**Keywords:** Short beak and dwarfism syndrome virus, Recombinase polymerase amplification, CRISPR/Cas12a, Dual readout, Onsite detection

## Abstract

Short beak and dwarfism syndrome virus (**SBDSV**) is the causative agent of short beak and dwarfism syndrome (**SBDS**), which is characterized by beak atrophy and dwarfism. SBDS has caused substantial economic losses for waterfowl husbandry industries. To address the urgent need for rapid and accurate SBDSV diagnostics, the study developed a dual-mode detection platform integrating recombinase-aided amplification (**RPA**) with CRISPR/Cas12a-mediated fluorescence and lateral flow strip readouts. The optimized assay achieved a detection limit of 10 copies/μL. Notably, the platform demonstrated superior specificity to distinguish SBDSV from genetically related Muscovy duck-origin goose parvovirus (**GPV**) and classical GPV, a distinction unachievable by qPCR. Clinical validation using 36 field samples confirmed 100% concordance with qPCR and indirect immunofluorescence assays, with no cross-reactivity against other common duck pathogens. This innovative detection system provides a robust toolkit for field-deployable SBDSV surveillance and lays a solid foundation for the development of novel diagnostic methodologies applicable to various waterfowl-related pathogens.

## Introduction

Goose parvovirus (**GPV**) is a nonenveloped, single-stranded DNA virus that poses a significant threat to the global waterfowl industry by causing diseases such as Derzsy's disease and short beak and dwarfism syndrome (**SBDS**) (D, 1967; Wang et al., 2015; Chen et al., 2016; Yan et al., 2019). The GPV genome, approximately 5.1 kb in length, comprises two major open reading frames (**ORFs**). The left ORF encodes two replication (**Rep**) proteins, Rep1 and Rep2 (Li et al., 2018), while the right ORF encodes three structural proteins (VP1- VP3) that constitute the viral capsid (Shao et al., 2020). Notably, the carboxyl terminal of VP1 encompasses all amino acid sequences of VP2 and VP3 (Tsai et al., 2004). Given that the VP3 coding sequence shows less variability compared to VP1 and VP2 (Tarasiuk et al., 2019), it is frequently targeted in GPV detection assays. GPV exhibits high genetic diversity and is categorized into classical GPV (**C-GPV**) and novel GPV (**N-GPV**), each of which is further divided into three distinct subgroups (Huo et al., 2023). This diversity is further compounded by frequent genetic recombination among GPV strains, influencing their adaptability and evolutionary (Huo et al., 2023). A prime example of this genetic diversity is the Muscovy duck-origin GPV (**MDGPV**), a recombinant strain derived from a natural recombination between GPV and Muscovy duck parvovirus (**MDPV**) (Wang et al., 2013; Liu et al., 2024). MDGPV is highly virulent in Muscovy ducklings, causing intestinal embolism similar to Derzy's disease (Wang et al., 2019; Wang et al., 2020). Another duck-origin GPV, SBDSV, which causes SBDS, originated from a GPV wild-type strain (Li et al., 2018). Recent SBDSV outbreaks in China has caused morbidity of 10% to 100% in Cherry Valley ducks, while in Egypt, outbreaks have resulted in 70% morbidity and 20% mortality in mule/Pekin ducks. These outbreaks have caused significant economic losses due to retarded growth and low performance in ducks (Chen et al., 2016; Soliman et al., 2020). Notably, SBDSV-associated feather shedding has been observed in both single infections and coinfections with duck circovirus (Yang et al., 2020). The virus has spread globally, affecting countries such as France, Poland, Egypt, and China. This underscores the urgent need for rapid diagnostic methods to control the transmission of SBDSV (Palya et al., 2009; Chen et al., 2016).

Traditional GPV detection methods encompass virus isolation (Chen et al., 2016), serological assays such as ELISA (Zhang et al., 2020), latex agglutination (**LA**) assay (Chen et al., 2016), and indirect immunofluorescence assays (**IFA**) (Chen et al., 2016). Although these techniques offer accuracy, they are labor-intensive and time-consuming. Nucleic acid amplification-based technologies such as conventional polymerase chain reaction (**PCR**) (Wozniakowski et al., 2010) and real-time quantitative polymerase chain reaction (**qPCR**) (Lin et al., 2019) have been employed for GPV detection. However, their reliance on heavy, power-intensive, and costly equipment makes onsite detection challenging. Although recombinase polymerase amplification (**RPA**) addresses some field limitations through rapid isothermal amplification (Chen et al., 2022; Wu et al., 2022; Bai et al., 2024), its susceptibility to aerosol contamination compromises reliability (Hu et al., 2022; Yin et al., 2024). Crucially, current methods fail to differentiate SBDSV from MDGPV due to their high nucleotide sequence similarity and inherent technical limitations. Moreover, both viruses are capable of infecting Muscovy ducks, often asymptptomatically, complicating clinical diagnosis. Consequently, there is an urgent demand for the development of a rapid, sensitive and field-portable visual detection method for SBDSV to ensure the sustainable and healthy development of the waterfowl industry.

An alternative approach to meet current requirements is the utilization of the CRISPR/Cas system, which has sparked a notable revolution in molecular diagnostics owing to its programmable ability to precisely cleave nucleic acids (Gootenberg et al., 2017; Li et al., 2018). In particular, the CRISPR effector Cas12a is known for its exceptional sensitivity and high specificity in targeting double-stranded DNA (**dsDNA**) or single-stranded DNA (**ssDNA**) sequences with a protospacer adjacent motif (**PAM**). Once triggered by guide RNA (**gRNA**), Cas12a not only cleaves the target DNA (cis-cleavage) but also indiscriminately cleaves any surrounding ssDNA (trans-cleavage) (Li et al., 2018; Zhang et al., 2024). This distinct feature enables the conversion of target nucleic acid sequences into detectable signals such as fluorescence and colorimetric values (Li et al., 2019). By utilizing reporters labeled with fluorophores and quenchers, the CRISPR/Cas system can release fluorescent signals upon cleavage. Alternatively, detection signals can be visualized via lateral flow dipsticks when the reporter is attached to biotin. Based on the above principles, CRISPR/Cas systems have been extensively utilized to detect various pathogens (Gootenberg et al., 2017; Li et al., 2018; Chen et al., 2023; Yang et al., 2024; Yin et al., 2024; Zhong et al., 2025). However, the application of the CRISPR/Cas method in SBDSV detection remains unexplored.

**Fig. 1:**
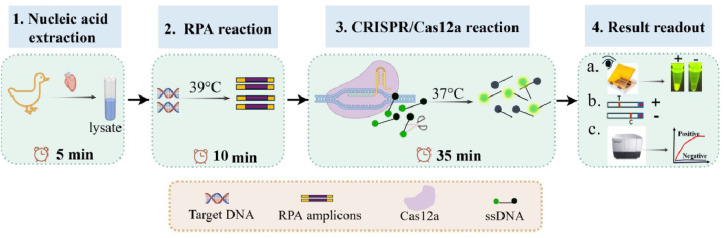
Schematic of the CRISPR/Cas12a diagnostic platform for GPV detection. The CRISPR/Cas12a diagnostic platform comprises four steps. First, tissue nucleic acids were extracted from the lysate. Second, nucleic acids are amplified in a 39°C metal bath for 10 min to ensure adequate target amplification. Third, the CRISPR/Cas12a system is employed to detect the RPA amplicons. Finally, the results can be interpreted using a portable blue light transilluminator, lateral flow strips, or a multimode microplate reader. Abbreviations: RPA, recombinase polymerase amplification; Cas12a, CRISPR-associated protein 12a; ssDNA, single-stranded DNA.

In this study, we combined the highly efficient amplification of RPA with the precise molecular sensing capability of Cas12a to establish an assay for the rapid detection of SBDSV. This assay yields both fluorescence detection and lateral flow assay (**LFA**) signal outputs and can be completed within one hour. Notably, the results can be visualized either with the naked eye under blue light or through lateral flow dipsticks, eliminating the need for DNA purification (Fig. 1); this makes the assay particularly suitable for low-resource settings.

To our knowledge, this work represents the first application of an RPA–CRISPR/Cas12a assay for the rapid, onsite visual detection of SBDSV, and it holds considerable promise for SBDSV screening in resource-constrained areas. The proposed assay may offer a novel avenue for establishing a robust diagnostic toolbox for managing various waterfowl diseases.

## Materials and methods

### Viral strains

SBDSV (GenBank KU844283.1), MDGPV(GenBank OQ301813.1), C-GPV (GenBank PQ272760.1), MDPV (GenBank KU844281.1), Muscovy duck reovirus (**MDRV,** GenBank GU369968.1), novel duck reovirus (**NDRV,** GenBank GQ888710.1), duck plague virus (**DPV**), duck adenovirus B2 (**DAdV B2,** GenBank PP763217.1), and duck tembusu virus (**DTMUV**) were isolated and preserved in our laboratory.

### Extraction of nucleic acids and generation of standard plasmids

The nucleic acids of the virus strains were extracted using the EasyPure® Viral DNA/RNA Kit (TransGen Biotechnology Co., Ltd., Beijing, China) following the manufacturer's instructions. To facilitate rapid detection, a nucleic acid rapid lysate (Shanghai Kanglang Biotechnology Co., Ltd., Shanghai, China) was employed to rapidly obtain crude extracts of nucleic acids from the tissues of clinical samples.

The *VP3* gene fragment was synthesized by Sangon Biotech and inserted into the pUC57 vector to construct the pUC57-VP3 plasmid. The copy number of the pUC57-*VP3* plasmid was determined via the following formula: [double-strand DNA copy number (copies/μL) = 6.02 × 10^23^ (copies/μL) × concentration (ng/μL)/DNA length × 660], where 660 denotes the average molecular weight of paired nucleotides. The copy number of the standard plasmid was determined to be 3.71 × 10^9^ copies/μL, which was subsequently 10-fold serially diluted to 3.71 × 10° copies/μL for further analysis.

### Design of the RPA primers and CRISPR RNA

To identify the most conserved regions suitable for targeting, complete genome sequences of the C-GPV, MDGPV, and SBDSV strains were retrieved from the NCBI database. These sequences were subsequently aligned via MegAlign in the DNASTAR program (version 7.1, DNASTAR). Based on the resulting alignment, a pair of RPA primers were designed to target the *VP3* gene using Primer Premier version 5.0 software (PREMIER Biosoft International, Palo Alto, California, USA). The CRISPR RNA (**crRNA**) was designed via an online tool (CHOPCHOP, https://chopchop.cbu.uib.no/). The BLAST program was then employed to assess the sequence conservation of the crRNA. The oligonucleotides used in this study were synthesized by Sangon Biotech (Shanghai, China). Detailed information regarding the designed primers and crRNA is provided in Table 1.

**Table 1:** Sequences of oligonucleotides used in this study.

Name	Lable	Sequence (5′to 3′)
RPA Primers	F1	TCAAGTCAAGGAAGTCACAACGCAGGAT
	F2	ATTTCAATCGCTTCCACTGCCACTTCTC
	R1	ATGGTCCCTTCCGTAGCCGAGCCCAGGA
	R2	ATTGAACCGTGCTCCATTCTGGTTGGTGTGC
	R3	TATGTCCTGGGCTCGGCTACGGAAGGGA
	crRNA*	UAAUUUCUACUAAGUGUAGAUCGGAUGAUGAGCACCAACUCCCGUAU
qPCR Primers	Q-F	GAGGTAGACAGCAACAGAAA
	Q-R	GCTCGTCCGTGACCATA
Probes	FQ-ssDNA	FAM-TTATT-BHQ1
	FB-ssDNA	FAM-TTTTTTTTATT-Biotin

⁎ The spacer sequences of the crRNA, which recognizes and complements the target, is labeled with an underline.

### RPA–CRISPR/Cas12a assay with fluorescence readouts

The platform provides two detection strategies, including a visual fluorescence-based system and a LFA-based detection. For the fluorescence-based approach, we followed a previously reported method with minor modification (Chen et al., 2023). Specifically, 100 nM LbCas12a protein (Guangzhou Magigen Biotechnology Co., Ltd., Guangzhou, China) and 200 nM crRNA were mixed in 10 × reaction buffer, followed by incubation at 37°C for 5 min to form the ribonucleoprotein (**RNP**) complex. Subsequently, 200 nM FQ-ssDNA probe, 1 μL RPA amplicons, and nuclease-free water were added to achieve a final volume of 30 μL. The mixture was immediately transferred to a 384-well black polystyrene microplate (Costar) and read using a multimode microplate reader (Bio Tek Synergy H1, Agilent Technologies, Inc., USA). Fluorescence signals were recorded every minute for 30 min, with excitation and emission wavelengths set at 485 nm and 525 nm, respectively. For visual detection, a portable blue light transilluminator (Tiangen Biotech Co., Ltd.) was employed, and results were captured with a smartphone camera (iPhone 8) in a darkened environment. To optimize detection performance, we meticulously adjusted the concentrations of crRNA, Cas12a, and the ssDNA reporter probe within the CRISPR/Cas12a system. To distinguish true target signals from nonspecific background noise, we utilized the signal-to-background (**S/B**) ratio as a metric for optimizing reaction conditions. The S/B ratio is defined as the fluorescence intensity in target-positive samples relative to that in no-template controls. Fluorescence intensity measurements were taken at the reaction endpoint (t = 30 min).

### RPA–CRISPR/Cas12a assay with lateral flow detection

In the LFA-based detection process, the first step was the same as the fluorescence assay. A reaction mixture containing 500 nM FB-ssDNA probe, 1 μL of RPA amplicons, and an appropriate amount of nuclease-free water was subsequently combined with the RNP complex solution to achieve a total volume of 20 μL. The positive control reaction utilized RPA amplicons derived from the positive plasmids, while non-target RPA amplicons served as the negative control. The mixture was then incubated at 37°C for 25 min to facilitate target-specific cleavage by the Cas12a RNP complex. Following incubation, the reaction mixture was supplemented with 80 μL of Tris-HCl buffer (0.05 M, pH 7.6). Commercial lateral flow dipsticks (Lesun Biotechnology Co., Ltd., Wuxi, China) were immersed in the reaction solution and incubated at room temperature for 5 min.The dipsticks were subsequently removed, and images were captured using a smartphone. SBDSV positivity was confirmed by the simultaneous appearance of both the test line (**T line**) and the control line (**C line**) on the dipstick. Conversely, only the C line appearing represented negativity for SBDSV.

### Evaluation of sensitivity and specificity

To evaluate sensitivity, 1 μL of serially diluted pUC57-VP3 plasmid ranging from 3.71 × 10^0^ to 3.71 × 10^3^ copies/μL was used as the template for the RPA. Subsequently, 1 μL of the RPA amplicons was used to activate the preassembled CRISPR/Cas12a reaction system. Nuclease-free ddH_2_O served as the template for the no-template control (**NTC**).

To examine specificity, nine nucleic acid samples, including SBDSV, MDGPV, C-GPV, MDPV, MDRV, NDRV, DPV, DAdV B2, and DTMUV, along with nuclease-free ddH_2_O, were tested.

### Application of the RPA–CRISPR/Cas12a assay to clinical samples

To validate the practical clinical feasibility of the CRISPR biosensing platform, it was employed to identify GPV infection in 36 deceased ducks from 8 farms across Putian, Zhangzhou and Longyan, Fujian Province (2021–2023). Cardiac tissue samples were collected within 2 h post-mortem to minimize nucleic acid degradation. The samples were preserved in DNA/RNA Buffer (New England Biolabs, China) and transported on dry ice to the laboratory within 6 h. Crude nucleic acid extractions were obtained using a rapid nucleic acid lysate method. The integrity of the extracted nucleic acids was validated via agarose gel electrophoresis and spectrophotometry. For long-term storage, the lysates were aliquoted and stored at −80°C until analysis. The crude extracts were then directly used for GPV detection via qPCR. Samples that tested positive were further subjected to RPA–CRISPR/Cas12a detection.

### Statistical analysis

Statistical analyses and figure generation were conducted via GraphPad Prism 8.0 software (GraphPad Software Inc., San Diego, CA, USA) and Origin 2019b software (OriginLab Corporation, Northampton, MA, USA). The differences between two groups were assessed via an unpaired Student’s t test. The experimental data are presented as the means ± SDs unless otherwise specified. N = three independent replicates, ^∗∗∗∗^ denotes P < 0.0001, ^∗∗^ represents P < 0.01, and ns indicates P > 0.05.

## Results and discussion

### Validation of method feasibility

**Fig. 2:**
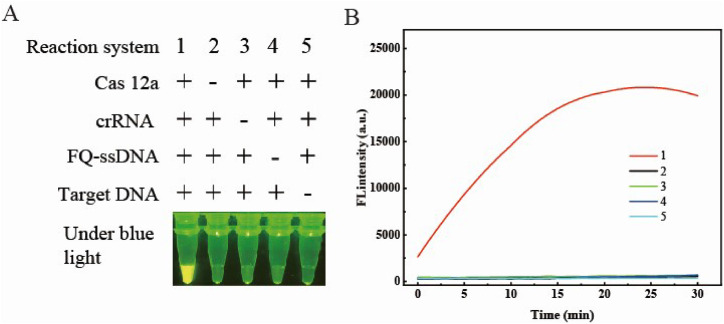
Feasibility analysis of the RPA–CRISPR/Cas12a assay. (A) Visual readouts of five reaction systems captured under blue light (470 nm). The symbols “+” and “−” denote the presence or absence, respectively, of the corresponding components in each reaction system. (B) Real-time fluorescence analysis of the same five reaction systems. The concentrations of Cas12a, crRNA, and the FQ-ssDNA probe were measured at 100 nM, 200 nM, and 300 nM, respectively. A plasmid containing the *VP3* gene of SBDSV was used as the target for the RPA assay, with the RPA amplicons subsequently serving as the target for the RPA–CRISPR/Cas12a assay.

The feasibility of the RPA–CRISPR/Cas assay for SBDSV detection was assessed by measuring the fluorescence intensity of five reaction systems, each comprising all components or lacking one. As illustrated in Fig. 2A, only reaction system #1 presented a strong fluorescence signal, whereas systems #2 to #5 presented no fluorescence signals. Similarly, Fig. 2B shows robust fluorescence in the presence of all components (curve 1), and the absence of any component resulted in an absence of fluorescence (curves 2 to 5). These results indicate the necessity of Cas12a, crRNA, and FQ-ssDNA probes for generating robust fluorescence signals. Moreover, the results confirmed the successful specific recognition of the RPA amplicons by the Cas12a–crRNA complex, which initiates the trans-cleavage activity of Cas12a and subsequent cleavage of the FQ-ssDNA probe.

### Screening of the optimal RPA primer pairs

Two forward primers and three reverse primers were designed to target the *VP3* gene of SBDSV. The validation of the most suitable RPA primers was carried out via agarose gel electrophoresis. As shown in Figure S1, the combination of the first forward primer and the second reverse primer produced a distinct and prominent 217 bp RPA product band without any nonspecific bands. Conversely, other primer combinations either resulted in faint bands or resulted in the presence of nonspecific amplified bands. Consequently, these primers were chosen for subsequent experiments.

### Complementarity validation of combining RPA with the CRISPR/Cas12a technique

**Fig. 3:**
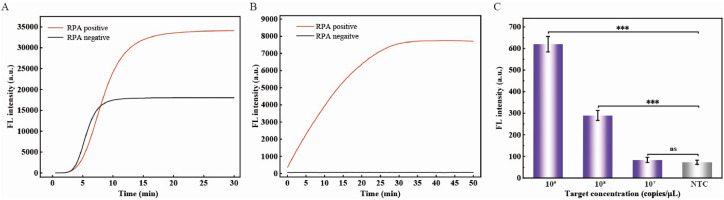
The necessity of integrating RPA with the CRISPR/Cas12a technique. (A) Fluorescence kinetics of RPA using plasmids with the *VP3* gene at a concentration of 10^5^ copies/μL (denoted as RPA positive) or nuclease-free ddH_2_O (denoted as RPA negative). EvaGreen dye was incorporated into the RPA reaction. (B) Real-time fluorescence dynamic curves of the CRISPR/Cas12a-based system using either RPA-positive amplicons or negative amplicons as targets. (C) Sensitivity of the CRISPR/Cas12a-based system without the aid of RPA. The data are presented as the means ± standard deviations (SDs) (where n = 3). Statistical significance is indicated as follows: “^⁎⁎⁎^” represents p < 0.001, whereas “ns” denotes a p value >0.05.

The nonspecific amplification phenomenon in RPA systems has been previously reported (Hu et al., 2022). To confirm this phenomenon, we compared the fluorescence intensities between a conventional RPA assay and an integrated RPA–CRISPR/Cas12a assay. As expected, the RPA negative control (target-free) exhibited robust fluorescence signals (Fig. 3A, black curve), indicating a significant risk of false-positive outcomes. This nonspecific amplification likely originates from primer-template mismatches or the high efficiency of RPA (Luo et al., 2019; Zhang et al., 2019). Notably, the fluorescence intensity of RPA reached a plateau at approximately 10 min, indicating highly efficient amplification of RPA. Therefore, we standardized the RPA reaction time to 10 min to balance amplicons yield and operational efficiency. Critically, integrating CRISPR/Cas12a with RPA suppressed nonspecific signal accumulation by more than 99% in negative controls (Fig. 3B), demonstrating the system’s enhanced specificity. This improvement is attributed to CRISPR/Cas12a’s sequence-specific collateral cleavage activity. Cas12a is activated only when it recognizes RPA amplicons containing both the target protospacer and the PAM sequence (Chen et al., 2018), thereby enabling the selective degradation of non-target products. To further evaluate the system, we measured the sensitivity of the CRISPR/Cas12a-mediated assay without RPA preamplification. The results revealed that the sensitivity was only 10^8^ copies/μL (Fig. 3C), which was consistent with previous reports (Ma et al., 2020; van Dongen et al., 2020; Chen et al., 2023). When RPA was used for preamplification of the *VP3* gene sequence, the sensitivity of the RPA–CRISPR/Cas12a assay was improved by 10^7^-fold (Fig. 5A). RPA enables rapid isothermal amplification of target DNA, elevating its concentration to detectable levels. CRISPR/Cas12a, guided by crRNA, specifically recognizes and binds to the amplified targets, triggering its collateral cleavage activity. This activity cleaves ssDNA at a rate of approximately 1,200 cuts per second, enabling exponential signal amplification (Chen et al., 2018). The synergy between RPA-mediated pre-amplification and Cas12a’s cascading signal amplification result in a 10⁷-fold enhancement in sensitivity. Therefore, the mild reaction temperature of the CRISPR/Cas12a system (approximately 37°C) and the efficiency of RPA technology can be combined to develop a method that integrates the complementary advantages of both methods approaches. This integration offers a promising strategy for enhancing both specificity and sensitivity in nucleic acid detection.

### Experimental condition optimization of the RPA–CRISPR/Cas assay

**Fig. 4:**
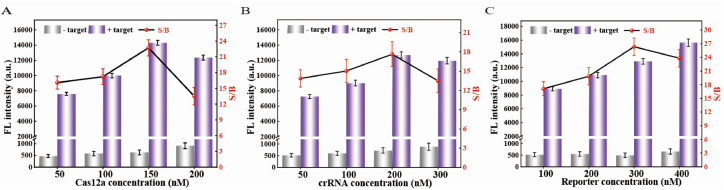
Optimization of the RPA–CRISPR/Cas12a assay conditions. Fluorescence intensity and S/B of the assay using different concentrations of Cas12a (A), crRNA (B), and the FQ-ssDNA reporter (C). The S/B represents the ratio between the fluorescence signal and background. The data are presented as the means ± standard deviations (SDs) (n = 3).

To improve the sensitivity of the assay, several crucial experimental conditions, including the concentrations of Cas12a, crRNA, and the FQ-ssDNA reporter probe, were optimized. In the CRISPR/Cas system, the speed of the shearing reaction is significantly influenced by the ratio of the Cas enzyme to the crRNA (Hu et al., 2022). Therefore, a series of Cas12a and crRNA concentrations were optimized to achieve optimal Cas12a cleavage efficiency. As depicted in Fig. 4A, increasing the Cas12a concentration from 50 nM to 150 nM yielded a substantial boost in end-point fluorescence intensity. However, escalating the concentration further to 200 nM resulted in a slight decline in fluorescence intensity alongside an elevated background signal, leading to a peak S/B ratio at 150 nM. A parallel optimization of crRNA concentration revealed a similar trend, with the S/B ratio peaking at 200 nM (Fig. 4B). This is consistent with previous reports indicating that an ample supply of gRNA facilitates the formation of the Cas12a ternary complex (Lv et al., 2021). Therefore, the optimized concentrations were determined to be 150 nM for Cas12a and 200 nM for crRNA. The concentration of the FQ-ssDNA reporter probe was subsequently further optimized. As shown in Fig. 4C, the fluorescence intensity gradually increased with the probe concentration rising from 100nM to 300 nM. However, an increase in the background signal was observed when the concentration reached 400 nM, which correspondingly reduced. Therefore, the final reporter concentration was set at 400 nM.

### Performance evaluation of the dual-mode RPA–CRISPR/Cas12a assay for detecting SBDSV

**Fig. 5:**
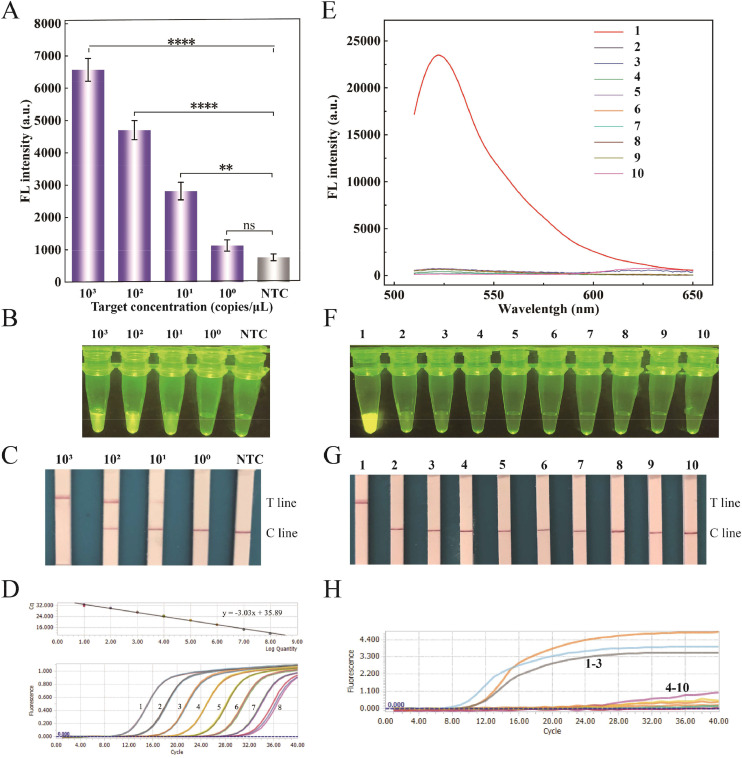
Performance assessment of the RPA–CRISPR/Cas12a assay. Sensitivity evaluation of the RPA–CRISPR/Cas12a assay by analyzing the end-point fluorescence (A), in-tube fluorescence visualization (B), and colorimetric lateral flow assay (C). NTC represents no template control. (D) Sensitivity of the quantitative real-time PCR assay. 1–8, represents 3.71 × 10^8^–3.71 × 10^1^ copies/μL of plasmids. All reactions were replicated three times. Specificity evaluation of the RPA–CRISPR/Cas12a assay by examining the fluorescence spectra of nine duck-origin viruses (E), in-tube fluorescence (F), and colorimetric lateral flow (G) assays. (H) Specificity of the quantitative real-time PCR assay. 1: short beak and dwarfism syndrome virus, 2: duck-origin goose parvovirus, 3: classical goose parvovirus, 4: Muscovy duck parvovirus, 5: Muscovy duck reovirus, 6: novel duck reovirus, 7: duck plague virus, 8: duck adenovirus B2, 9: duck tembusu virus, 10: ddH_2_O. All viral nucleic acids were present at the same concentration. The data are presented as the means ± standard deviations (SDs) (n = 3). Statistical significance is denoted as follows: “^⁎⁎⁎⁎^” indicates p < 0.0001, “^⁎⁎^” signifies p < 0.01, and “ns” denotes a p value > 0.05.

To evaluate the sensitivity of the optimized RPA–CRISPR/Cas12a assay, a series of 10-fold serial dilutions of recombinant plasmids, ranging from 10^0^ to 10^3^ copies/μL, were employed as target DNA for the RPA reaction. Subsequently, 1 μL of the RPA amplicons was introduced into the CRISPR/Cas12a system. As depicted in Fig. 5A, the fluorescence-based RPA–CRISPR/Cas12a assay achieved a detection limit of 10 copies/μL, demonstrating a 10^7^-fold improvement in sensitivity compared to methods without RPA preamplification (Fig. 3C). These results were further validated through visual observation under 470 nm blue light (Fig. 5B). The assay's sensitivity was comparable to that of the qPCR assay (Fig. 5D) and outperformed all other GPV detection methodologies (Table 2). This exceptional sensitivity can be attributed to the robust amplification capabilities of RPA and the high cleavage efficiency of CRISPR/Cas12a system (Chen et al., 2023). Unlike conventional GPV detection methods, the proposed assay eliminates the need for complex instrumentation and allows for visual readout (Table 2).

**Table 2:** Comparison of our established method with previous methods for GPV detections.

Strategy	Target	LOD	Total time	Visualization	Required instruments	Differentiate SBDSV from MDGPV	Ref.
Virus isolation and electron microscopy	SBDSV	-	6-7 days	No	CO2 incubator and electron microscopy	No	Chen et al., 2016
IFA a	SBDSV	-	∼90 min	No	Freezing microtome and fluorescence microscope	No	Chen et al., 2016
Conventional PCR	C-GPV	2 TCID50 b	∼150 min	No	PCR thermal cycler and gel imager	No	Wozniakowski et al., 2010
TaqMan based qPCR	SBDSV	10^2^ copies	> 60 min	No	qPCR thermal cycler	No	Wang et al., 2017
RPA-CRISPR/Cas12a	SBDSV	3.71 × 10^1^ copies	50 min	Yes	Heat bath and blue light device	Yes	This work

a IFA, indirect immunofluorescence assay

b TCID50, tissue culture infective dose 50

To simplify equipment requirements and enhance result interpretation, we developed an RPA–CRISPR/Cas12a assay integrated with a lateral flow strip for colorimetric output. As expected, the RPA–CRISPR/Cas12-LFS method exhibited sensitivity comparable to both end-point fluorescence detection and visual fluorescence detection (Fig. 5C).

The analytical specificity of the assay was evaluated by testing various duck-origin viruses, including SBDSV, MDGPV, C-GPV, MDPV, MDRV, NDRV, DPV, DAdV B2, and DTMUV. Results showed that the fluorescence-based RPA–CRISPR/Cas12a assay produced a robust fluorescence signal exclusively for SBDSV, while other duck-origin viruses and the negative control exhibited no fluorescence (Fig. 5E). Similarly, only SBDSV displayed a discernible green fluorescence signal and a clear T line on the lateral flow strip (Fig. 5F and G), whereas others showed faint fluorescence signals and only a C line. In contrast, the qPCR assay detected three types of GPV (SBDSV, MDGPV, and C-GPV), highlighting its limited differentiation capability among these closely related viruses (Fig. 5H). Given that SBDSV, MDGPV, and C-GPV share nearly identical nucleotide sequences, accurately distinguishing among them using conventional diagnostic methods such as conventional PCR, multiplex PCR, and qPCR is challenging. In contrast, our assay effectively differentiates these viruses. This highlights the remarkable selectivity of the developed CRISPR/Cas12a assay for SBDSV. The high specificity of CRISPR/Cas12a arises from its PAM recognition and R-loop formation dynamics. Single-base mismatches in critical regions can prevent R-loop completion, blocking target activation and subsequent collateral cleavage. This multi-step interrogation mechanism minimizes off-target effects and enhances specificity (Aris et al., 2025). This precise recognition mechanism allows the CRISPR/Cas12a system to distinguish between closely related viruses with high fidelity, making it a powerful tool for accurate viral detection. Overall, the dual-mode RPA–CRISPR/Cas12a assay not only matches the sensitivity of qPCR but also offers superior selectivity for SBDSV, making it a promising method for precise detection of SBDSV in clinical settings.

### Field-deployable detection of SBDSV with the dual-mode RPA–CRISPR/Cas12a assay

**Fig. 6:**
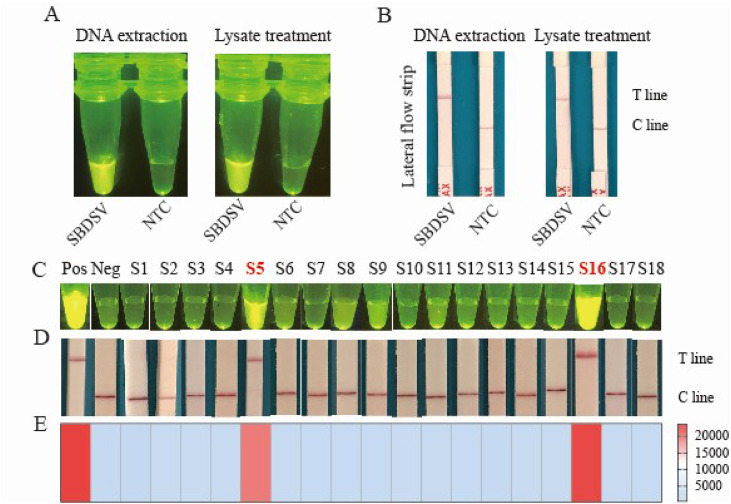
Application of the RPA–CRISPR assay for testing SBDSV in clinical samples. Feasibility of using nucleic acid rapid lysates through an end-point fluorescence visualization strategy (A) and lateral flow strips (B). (C) Visualized fluorescence signal detection under blue light following a 20-minute incubation period. These images were subsequently captured via a smartphone camera. (D) Cas12a-based lateral flow of 18 clinical samples. (E) Heatmap of the end-point fluorescence of 18 clinical samples. Pos represents reactions containing SBDSV nucleic acid, whereas Neg represents reactions devoid of SBDSV nucleic acid. The samples are labeled from S1 to S18. Notably, positive results are indicated by a red color, whereas negative results are denoted in blue.

Efficient sample preparation is critical for accurate clinical sample detection. In this study, a nucleic acid extraction reagent was used to rapidly generate suitable extracts (containing total nucleic acids) from clinical samples within just 5 min. This streamlined method eliminates the need for laborious, multi-reagent protocols typically associated with viral DNA/RNA extraction kits. To assess the suitability of these crude lysate for direct visual detection, we applied the RPA–CRISPR/Cas assay on two clinical samples (one positive and one negative) both with and without prior DNA purification. Notably, the SBDSV-positive samples exhibited equally intense green fluorescence signals regardless of DNA purification, whereas the SBDSV-negative samples showed faint fluorescence signals (Fig. 6A). Furthermore, when using the lateral flow detection strategy, SBDSV-positive samples consistently displayed a clear C line, regardless of the nucleic acid extraction method, while the negative control showed a T line (Fig. 6B). These results demonstrate that crude lysate extracts are sufficiency for visually distinguishing between positive and negative samples, significantly simplifying the testing workflow.

Based on these findings, we assessed the reliability of the dual-mode RPA–CRISPR/Cas12a assay using 36 deceased ducks suspected of having GPV infection, characterized by dysplastic feathers (Figure S3A) and pancreatic white spots (Figure S3B). Among these samples, 18 were confirmed GPV positive via qPCR (Figure S2) and were further tested with our RPA–CRISPR/Cas12a assay. The assay successfully identified two samples (S5 and S16) that emitted bright green fluorescence signals (Fig. 6C), while the remaining 16 samples showed no visible fluorescence. When integrated with lateral flow strip, the RPA–CRISPR/Cas12a system accurately identified the two positive samples (S5 and S16), confirming the fluorescence findings (Fig. 6D). Subsequent end-point fluorescence analysis further validated the SBDSV positivity of these samples (Fig. 6E). Notably, all results were consistent with those obtained from IFA (Figure S4). Compared to other molecular detection techniques, the proposed RPA–CRISPR/Cas12a approach offers several advantages such as a constant reaction temperature of 37°C, simplicity, and rapidity in producing visualization results (as shown in Table 2). Additionally, unlike other methods, our assay can specifically identify SBDSV and MDGPV. These results demonstrate the high reliability and feasibility of the RPA–CRISPR/Cas12a assay for SBDSV detection in clinical samples.

## Conclusion

In this study, a dual-mode detection assay integrating the RPA and CRISPR/Cas12a systems was successfully developed for the rapid, sensitive, and portable detection of SBDSV. The assay exhibits exceptional specificity, accurately distinguishing SBDSV from related pathogens, and achieved a remarkable sensitivity with a detection limit of 10 copies/μL. Notably, the results are discernible to the naked eye without any sophisticated instruments, making it highly suitable for field detection. We validated the assay's effectiveness by achieving robust and accurate detection of SBDSV in clinical samples within 50 min. Despite the significant advancements represented by this assay, there remains room for further optimization. One potential improvement involves integrating the RPA and CRISPR/Cas12a components into a single tube, which could streamline workflows and reduce the risk of aerosol contamination. Additionally, the implementation of precise nucleic acids quantification would be beneficial for monitoring disease progression and evaluating the effectiveness of intervention strategies. Given the high prevalence of co-infections on farms, there is also an urgent need to develop multiplex assays capable of detecting multiple pathogens simultaneously, which would greatly enhance overall diagnostic capacity.

In conclusion, the dual-mode RPA-CRISPR/Cas12a assay developed in this study holds substantial promise for the clinical testing of SBDSV. Its rapid, sensitive, and user-friendly nature makes it a valuable tool in the fight against SBDSV, particularly in resource-limited settings. As we continue to refine and expand this technology, we expect it to play a pivotal role in the sustainable management of waterfowl diseases.

## Funding

This research was funded by grants sponsored by the Scientific Research Project of Fujian Academy of Agricultural Sciences (Grant No. ZYTS2023017), the Fujian Public Welfare Project (Grant No. 2023R1024002, 2023R1024003, 2022R1026004) and the Central Government Guides Local Scientific and Technological Development Project (Grant No. 2022L3019).

## Ethics approval and consent to participate

All experimental procedures were reviewed and approved by the Institute of Animal Husbandry and Veterinary Medicine, Fujian Academy of Agricultural Science Animal Care and Use Committee (license number: MYLLSC2024-007).

## Declaration of competing interest

The authors declare the following financial interests/personal relationships which may be considered as potential competing interests: Xiuqin Chen, Su Lin, Shao Wang reports financial support was provided by Fujian Province Department of Science and Technology. Shilong Chen reports financial support was provided by Ministry of Science and Technology of the People’s Republic of China. Reports a relationship with that includes:. Has patent pending to. If there are other authors, they declare that they have no known competing financial interests or personal relationships that could have appeared to influence the work reported in this paper.

## Appendix Supplementary materials

Image, application 1

Image, application 2
