# Supplementary material for: From lab to field: Innovative RPA‒CRISPR/Cas12a platform for early short-beak and dwarfism syndrome virus nucleic acids detection
Source: Poult Sci. 2025 Apr 19;104(7):105191. doi: 10.1016/j.psj.2025.105191 (PMC12051591; doi:10.1016/j.psj.2025.105191)
Supplement: Supplementary file 1 [file mmc1.pdf]

Editing Certificate

This document certifies that the manuscript

From Lab to Field: Innovative RPA-CRISPR Platform for Early Short Beak and Dwarfism Syndrome Virus Detection

prepared by the authors

Xiuqin Chen, Shizhong Zhang, Su Lin, Meiqing Huang, Shao Wang, Shilong Chen

was edited for proper English language, grammar, punctuation, spelling, and overall style by one or more of the highly qualified English speaking editors at AJE.

This certificate was issued on **January 2, 2025** and may be verified on the AJE website using the verification code **A0A3-CEA0-0A0F-F33D-1F0A**.

Neither the research content nor the authors' intentions were altered in any way during the editing process. Documents receiving this certification should be English-ready for publication; however, the author has the ability to accept or reject our suggestions and changes. To verify the final AJE edited version, please visit our verification page at aje.com/certificate. If you have any questions or concerns about this edited document, please contact AJE at support@aje.com.

AJE provides a range of editing, translation, and manuscript services for researchers and publishers around the world. For more information about our company, services, and partner discounts, please visit aje.com.
