# Supplementary material for: From lab to field: Innovative RPA‒CRISPR/Cas12a platform for early short-beak and dwarfism syndrome virus nucleic acids detection
Source: Poult Sci. 2025 Apr 19;104(7):105191. doi: 10.1016/j.psj.2025.105191 (PMC12051591; doi:10.1016/j.psj.2025.105191)
Supplement: Supplementary file 2 [file mmc2.docx]

**From lab to field: Innovative RPA‒CRISPR/Cas12a platform for early** **detection of short-beak and dwarfism syndrome virus nucleic acids**

Xiuqin Chen^a, b^, Shizhong Zhang^a^, Su Lin^a, b^, Meiqing Huang^a, b^, Shilong Chen^a, b^, Shao Wang^a, b*^

^a^ Institute of Animal Husbandry and Veterinary Medicine, Fujian Academy of Agricultural Science, Fuzhou, Fujian 350013, China；

^b.^ Fujian Animal Diseases Control Technology Development Center, Fuzhou, Fujian 350013, China.

***Corresponding author**E-mail: wangshao@faas.cn

**Supplementary Methods**

**RPA assay**

The RPA assay was performed using the TwistAmp® Basic Kit (TwistDx Ltd., Cambridge, UK) following the manufacturer's protocol with slight modifications. Briefly, 29.5 μL of rehydration buffer, 480 nM of each forward and reverse primer, and nuclease-free ddH_2_O were added to a lyophilized RPA reaction pellet to reach a final volume of 46.5 μL. For cost efficiency, this solution was equally divided into two tubes. Then, 1 μL of the plasmid standard and 1.25 μL of 280 nM magnesium acetate were added to each tube and mixed thoroughly. The reaction tubes were then incubated at 39 °C in a metal heater for 10 min. The resulting amplicons were subjected to RPA–CRISPR/Cas12a analysis.

To determine the nonspecific amplification of the RPA reaction, 20×EvaGreen dye (Biotium, CA, China) was incorporated into the RPA reaction system. The RPA assay was conducted at 39 °C for 30 min, with real-time monitoring of the fluorescence curves.

**Quantitative real-time PCR assay**

The qPCR assay for GPV detection was conducted as described previously (Lin et al., 2019) via a Roche LightCycler® 96 instrument (Roche Diagnostics, Germany). A plasmid containing VP3 was subjected to 10-fold serial dilutions, ranging from 3.71× 10^1^ to 3.71 × 10^8^ copies/μL. Each dilution was used as a template for the qPCR analysis. The qPCR mixture consisted of 10 μL of PerfectStart® Green qPCR SuperMix (TransGen Biotechnology Co., Ltd., Beijing, China), 0.5 μL of each forward and reverse primer (10 μM), 1 μL of DNA template, and 8 μL of nuclease-free ddH_2_O, resulting in a final volume of 20 μL. The reaction procedure involved initial denaturation at 95 °C for 5 min, followed by 40 cycles of 95 °C for 15 s, 60 °C for 10 s, and 72 °C for 15 s. Samples with a cycle threshold (Ct) value below 35.0 and a melting temperature (Tm) of 86.15 ± 0.26 °C were considered positive for GPV.


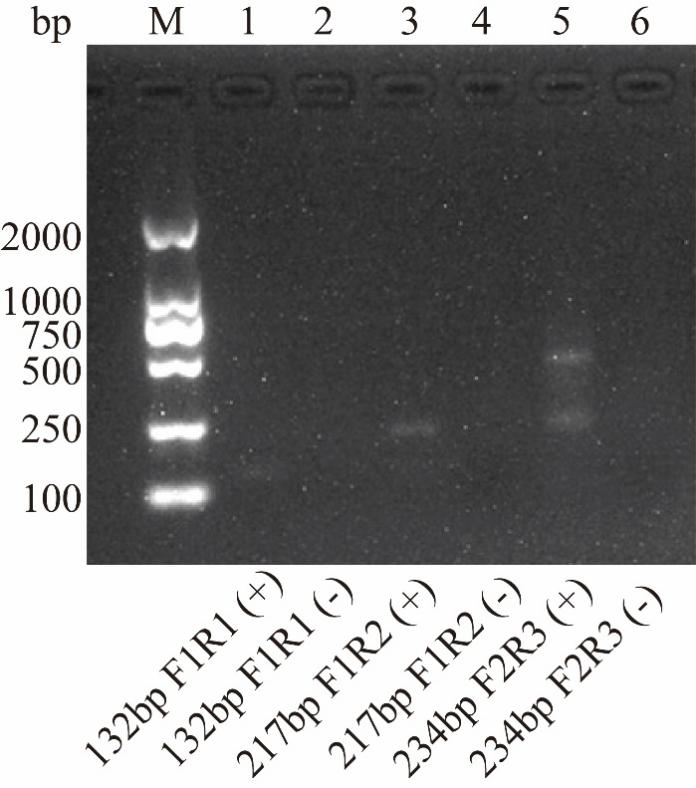


Figure S1 Optimization of RPA primer pairs for screening SBDSV. Optimization was conducted via agarose gel electrophoresis (2%) using standard plasmids at a concentration of 10^5^ copies/μL as the template. Lane M: DL2000 marker; the odd-numbered lanes are amplified bands obtained from the F1R1, F1R2 and F2R3 combinations (containing templates). The even numbered lanes are negative controls for the corresponding primer combinations. The expected sizes of the RPA products for the F1R1, F1R2 and F2R3 combinations were 132 bp, 217 bp, and 234 bp, respectively, aligning with the expected sizes outlined in Table 1.


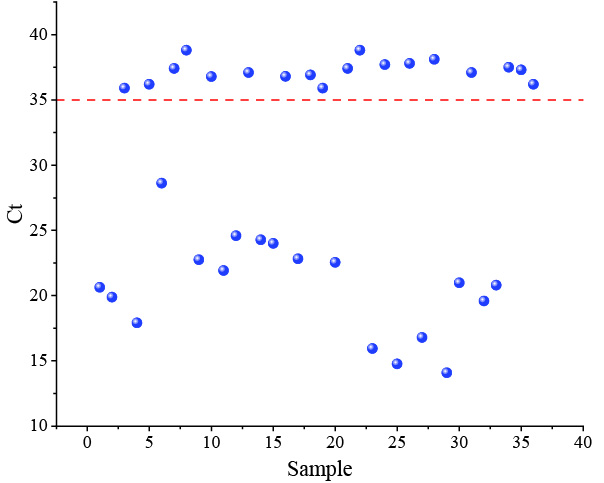


Figure S2 Results of the qPCR assay of clinical samples. A Ct value of less than 35 indicates a positive sample, whereas a Ct value greater than 35 denotes a negative sample.


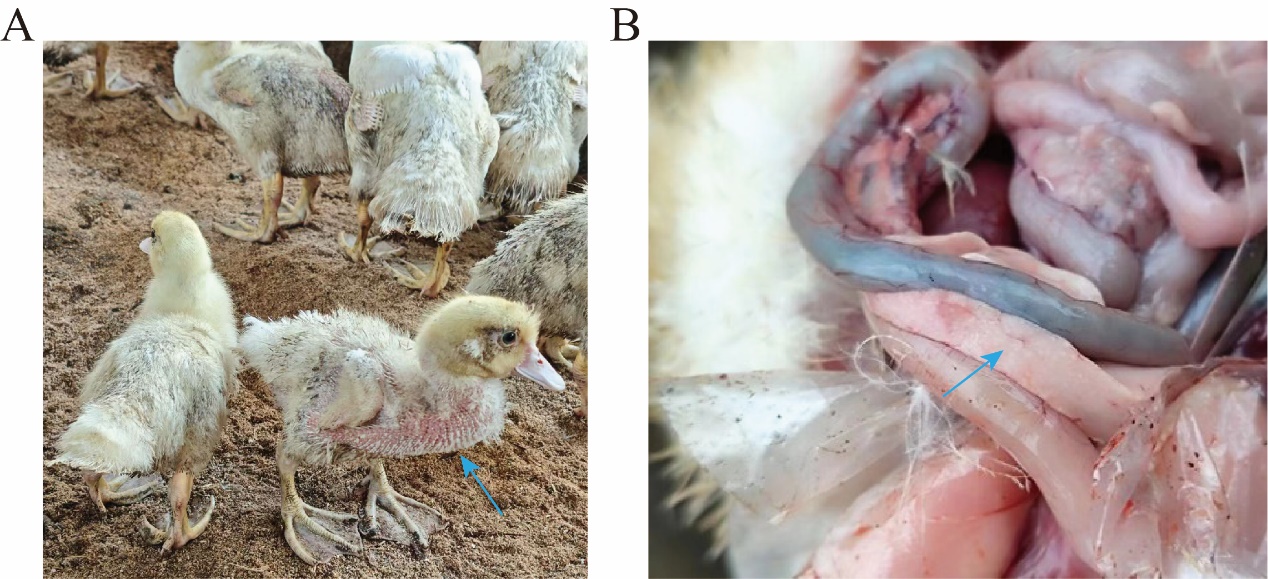


Figure S3 Clinical symptoms and pathological changes observed in diseased ducks from the clinical breeding farm. (A) Dysplastic feathers; (B) Pancreatic white spots.


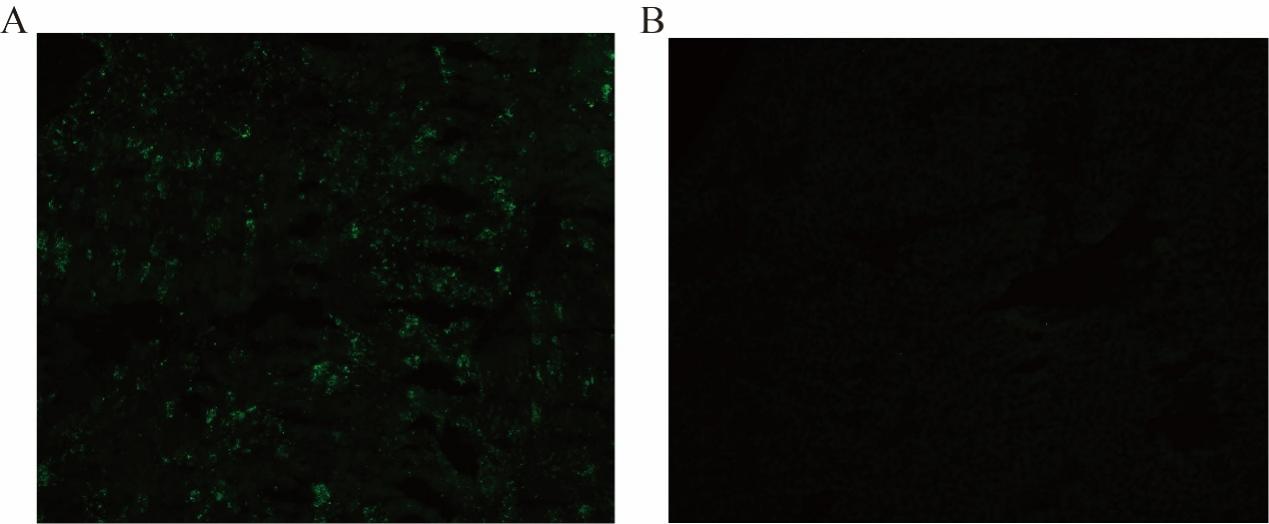


Figure S4 Indirect immunofluorescence assay for the detection of GPV antigen (×40). The primary antibody used was MAb E16, specific to GPV, and the secondary antibody was FITC-conjugated goat anti-mouse immunoglobulin. (A) Positive clinical samples exhibiting fluorescence; (B) Negative clinical samples showing no fluorescence.

**Reference**

Lin, S., S. Wang, X. Cheng, S. Xiao, X. Chen, S. Chen, S. Chen, and F. Yu. 2019. Development of a duplex SYBR Green I-based quantitative real-time PCR assay for the rapid differentiation of goose and Muscovy duck parvoviruses. Virol J 16(1):6.
